# Supplementary figures and images for: Genomic analysis of the diversity, antimicrobial resistance and virulence potential of clinical Campylobacter jejuni and Campylobacter coli strains from Chile
Source: PLoS Negl Trop Dis. 2021 Feb 19;15(2):e0009207. doi: 10.1371/journal.pntd.0009207 (PMC7928456; doi:10.1371/journal.pntd.0009207)

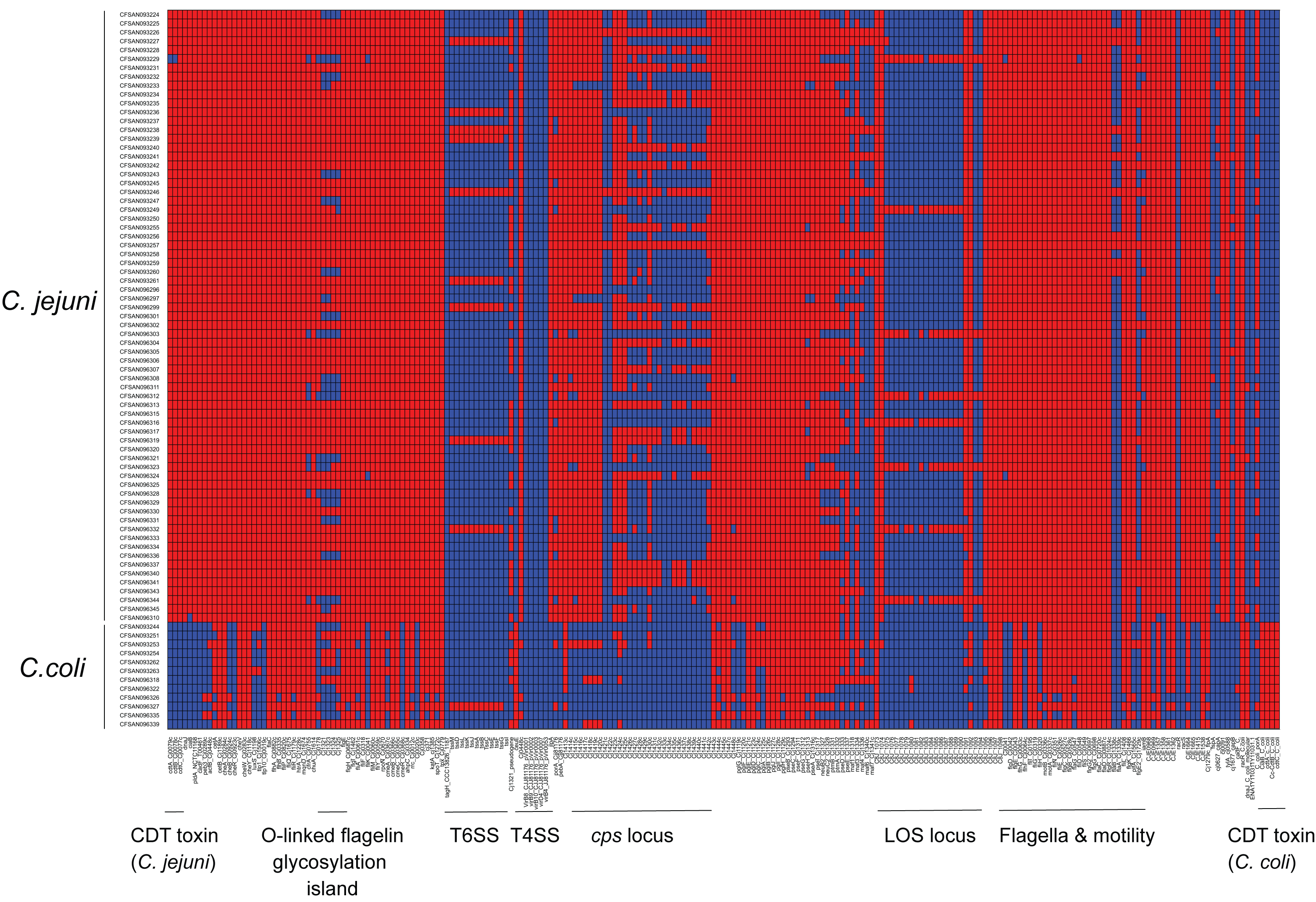

Supplement: S1 Fig — (TIF) [file pntd.0009207.s001.tif]

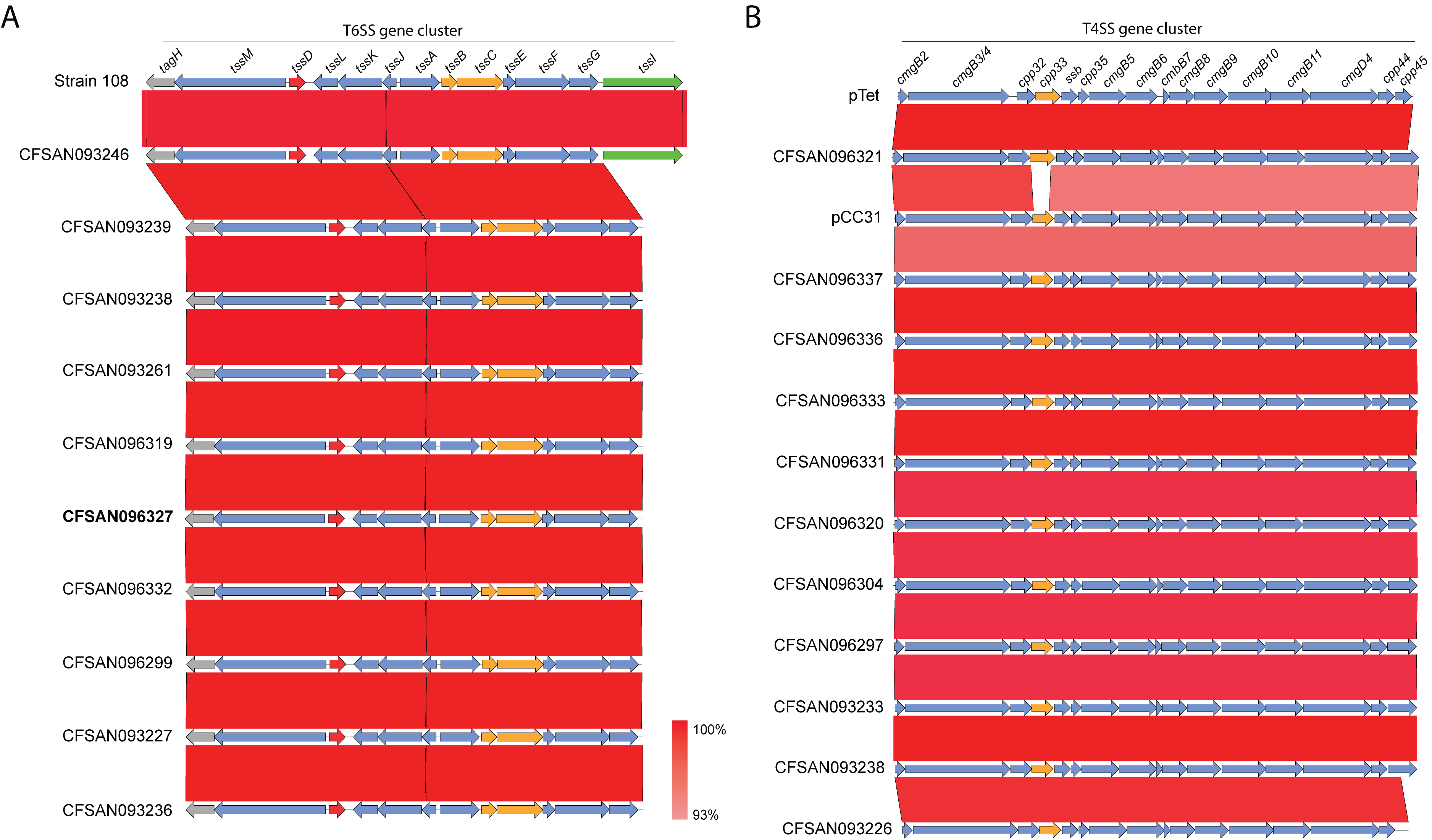

Supplement: S2 Fig — (A) T6SS gene clusters of C. jejuni and C. coli strains (bold) compared to the cluster of C. jejuni strain 108. Genes tagH, tssD, tssB and tssC are shown in color. (B) T4SS gene clusters of C. jejuni strains in comparison to the T4SS gene clusters of pTet and pCC31. The cp33 gene is highlighted in orange. BLASTn alignments were performed and visualized using EasyFig. (TIF) [file pntd.0009207.s002.tif]
